# Fine mapping and candidate gene analysis of the white flower gene *Brwf* in Chinese cabbage (*Brassica rapa* L.)

**DOI:** 10.1038/s41598-020-63165-7

**Published:** 2020-04-08

**Authors:** Ning Zhang, Lin Chen, Shuai Ma, Ruofan Wang, Qiong He, Min Tian, Lugang Zhang

**Affiliations:** 1 State Key Laboratory of Crop Stress Biology for Arid Areas, College of Horticulture, Northwest A&F University, Yangling, 712100 Shaanxi China; 2 State Key Laboratory of Vegetable Germplasm Innovation, Tianjin, 300384 China

**Keywords:** Plant breeding, Plant genetics

## Abstract

Flower color can be applied to landscaping and identification of the purity of seeds in hybrid production. However, the molecular basis of white flower trait remains largely unknown in *Brassica rapa*. In this study, an F_2_ population was constructed from the cross between 15S1040 (white flower) and 92S105 (yellow flower) for fine mapping of white flower genes in *B*. *rapa*. Genetic analysis indicated that white flower trait is controlled by two recessive loci, *Brwf1* and *Brwf2*. Using InDel and SNP markers, *Brwf1* was mapped to a 49.6-kb region on chromosome A01 containing 9 annotated genes, and among them, *Bra013602* encodes a plastid-lipid associated protein (PAP); *Brwf2* was located in a 59.3-kb interval on chromosome A09 harboring 12 annotated genes, in which *Bra031539* was annotated as a *carotenoid isomerase* gene (*CRTISO*). The amino acid sequences of BrPAP and BrCRTISO were compared between two yellow-flowered and three white-flowered lines and critical amino acid mutations of BrPAP and BrCRTISO were identified between yellow-flowered and white-flowered lines. Therefore, *Bra013602* and *Bra031539* were predicted as potential candidates for white flower trait. Our results provide a foundation for further identification of *Brwf* and increase understanding of the molecular mechanisms underlying white flower formation in Chinese cabbage.

## Introduction

In nature, flower color was used to attract insect for pollination in plants^1^. There are three chemically distinct pigments, carotenoids, flavonoids, and betalains, responsible for flower color, and among them, carotenoids accumulating in petals can generate yellow, orange, and red flower colors^2,3^. The most common carotenoids in petals are xanthophylls, which show high specificity in composition and quantity among plant species or varieties^4^.

Carotenoid accumulation was modulated by its biosynthesis, degradation, and sequestration^5–8^. The mutation of key genes involved in the above three processes could result in the conversion of flower and fruit colors. For example, a single-nucleotide mutation in *β-carotene hydroxylase 2* (*CHYB2*) caused orange fruit phenotype in pepper^9^. In *Chrysanthemum morifolium*, *Brassica napus*, and *B*. *oleracea*, the loss-of-function mutation of *carotenoid cleavage dioxygenase 4* (*CCD4*) led to change in flower color from white to yellow^7,10–14^. The mutation of *pale yellow petal* (*PYP1*) that was involved in xanthophyll ester production was responsible for pale yellow petal phenotype in tomato^8^.

The *Brassica* genus includes important oil crops and vegetables^15^ with yellow flower color as the most common form, while there are other colors, such as pale yellow, white, orange, and tangerine^16–22^. Compared with the studies of other traits in *Brassica* genus, such as yield^23–28^, fertility^29–32^, disease resistance^33–35^, the genetic studies of flower colors have been conducted earlier^16,17,36^. However, only the molecular mechanisms of white flower formation were understood^11–14,37,38^. Recently, a few genes controlling flower colors have been reported. For example, a *carotenoid isomerase* gene (*CRTISO*) related to pale yellow flower in *B*. *rapa*^22^ and a *carotenoid cleavage dioxygenase 4* gene (*CCD4*) associated with white flower in *B*. *napus*^11^ and *B*. *oleracea*^12–14^ were cloned, respectively. In *B*. *napus* and *B*. *oleracea*, a CACTA-like transposable element insertion caused disruption of *CCD4*^11,13,14^ and *CCD4* from white-flowered line could rescue the petal color of yellow-flowered line^11,12^. In addition, Zhang *et al*.^37,38^ reported that the *BjuA008406* and *BjuB027334* genes, which might be involved in carotenoid esterification, were predicted as the potential candidates for white flower in *B*. *juncea*. However, none of white flower genes has been cloned yet, and the molecular mechanism of white flower formation remains poorly understood in *B*. *rapa*.

In this study, the inheritance pattern of white flower trait was analyzed using an F_2_ segregating population developed from the crossing of white flower line 15S1040 and yellow flower line 92S105. Molecular markers designed based on the genome re-sequencing data of 15S1040 and 92S105 were used to map white flower genes, and then the prediction of the candidate genes was performed; the coding sequences of two candidate genes (*BrPAP* and *BrCRTISO*) were compared between three white-flowered and two yellow-flowered lines; the expression levels of two candidate genes were tested in different tissues. Our findings provide insights in molecular mechanisms controlling flower color variation in *B*. *rapa*.

## Results

### Genetic analysis of the white flower trait in *B*. *rapa*

**Figure 1:**
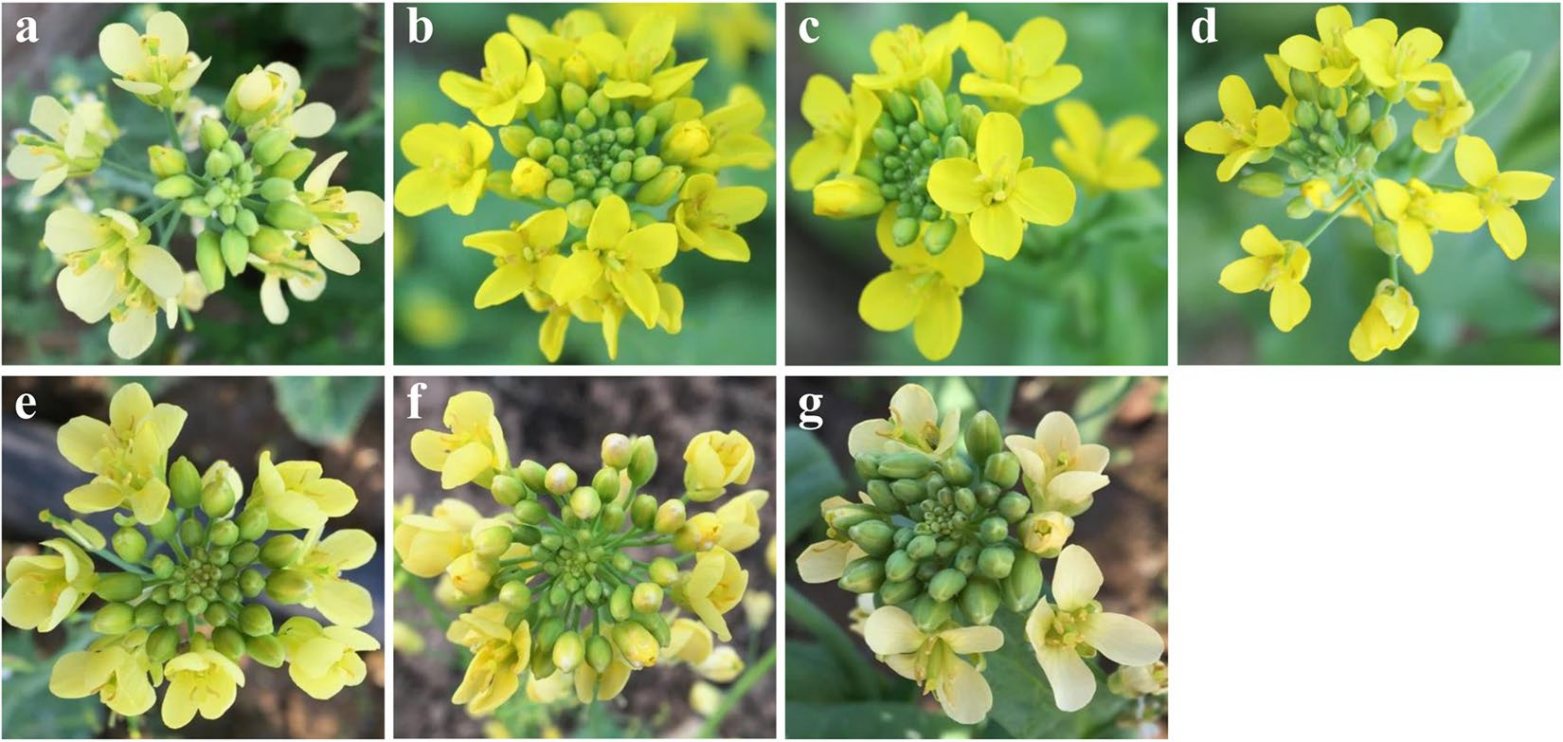
Flower colors of the two parents and their F_1_ (**c**) and F_2_ (**d–g**) generations. (**a**) 15S1040, (**b**) 92S105; (**c**) F_1_ individual; (**d**) Yellow flower F_2_ individual, (**e**) Milky yellow flower F_2_ individual, (**f**) Pale yellow flower F_2_ individual, (**g**) White flower F_2_ individual.

The flower colors of F_1_ plants derived from the cross between white parent 15S1040 and yellow parent 92S105 were all yellow (Fig. 1a–c). Among 1282 F_2_ individuals, 718 individuals were yellow flower, 257 individuals were milky yellow flower, 227 individuals were pale yellow flower, and 80 individuals were white flower (Fig. 1d–g). The F_2_ segregation ratio was fitted into an expected ratio of 9:3:3:1 (χ^2^ = 1.908, *df* = 3, *P* > 0.05) using *χ*^2^ test (Table 1). These results indicated that yellow flower trait was dominant over white flower and the white flower trait was controlled by two recessive genes, *Brwf1* and *Brwf2*, therefore the genotypes of four flower color plants may be yellow flower (*BrWF1BrWF1BrWF2BrWF2*, *BrWF1BrWF1BrWF2Brwf2*, *BrWF1Brwf1BrWF2BrWF2*, or *BrWF1Brwf1BrWF2Brwf2*), milky yellow flower (*Brwf1Brwf1BrWF2BrWF2* or *Brwf1Brwf1BrWF2Brwf2*), pale yellow flower (*BrWF1BrWF1Brwf2Brwf2* or *BrWF1Brwf1Brwf2Brwf2*) and white flower (*Brwf1Brwf1Brwf2Brwf2*), respectively.

**Table 1:** The segregation of flower colors in the F_1_ and F_2_ population.

Cross combination	Generation	Total plants	Yellow flower plants	Milky yellow flower plants	Pale yellow flower plants	White flower plants	Mendelian expectation	χ^2^ value^a^(*df* = 3, *P* > 0.05)
15S1040×92S105	F_1_	20	20					
	F_2_	1282	718	257	227	80	9:3:3:1	1.908

^a^ χ^2^ > χ^2^_(0.05, 3)_ = 7.815 is considered significant.

### Carotenoid accumulation and ultrastructural analysis of chromoplasts in yellow and white petals

**Figure 2:**
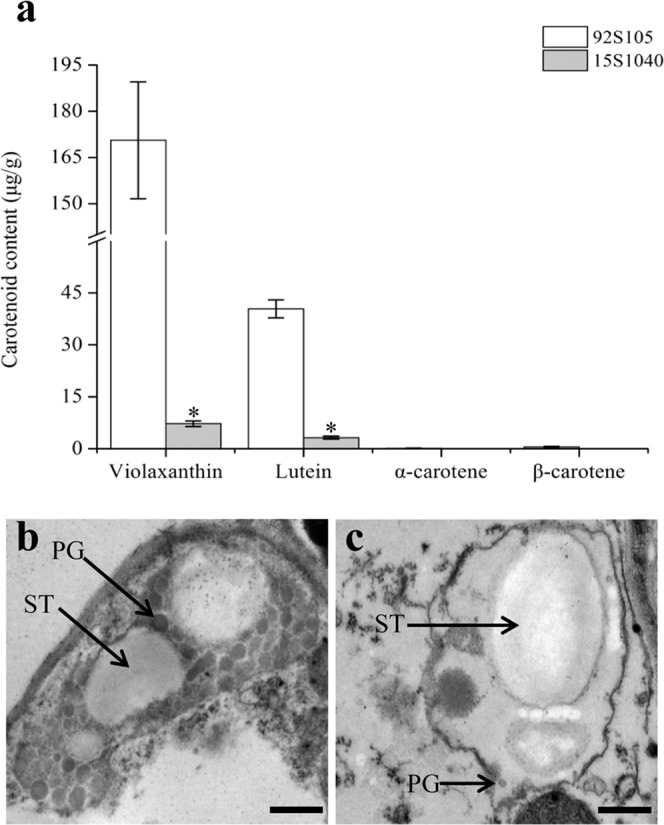
Carotenoid composition and content and chromoplast ultrastructure in anthesis petals of 92S105 and 15S1040. (**a**) Carotenoid composition and content of 92S105 and 15S1040. Error bars indicate the standard deviation (SD), and asterisks represent significant difference (t-test, *P* < 0.05) between 92S105 and 15S1040. (**b**) Chromoplast morphology in the petal of 92S105. (**c**) Chromoplast morphology in the petal of 15S1040. PG: plastoglobule, ST: starch, bar= 0.5 μm (**b**,**c**).

Carotenoid composition and content in yellow and white petals at the flowering stage were analyzed using high performance liquid chromatography (HPLC). The results showed that the major carotenoids in yellow and white petals were both violaxanthin and lutein, however, the total carotenoid contents of yellow and white petals were 211.69 ± 21.70 μg/g and 10.49 ± 1.21 μg/g (Fig. 2a), respectively, which may result in the difference in color between yellow and white petals.

To study whether there were differences in chromoplast structures between yellow and white petals, the ultrastructural analysis of chromoplasts in the two parents was performed using transmission electron microscopy (TEM). The results indicated that yellow-flowered individuals had normal chromoplasts with numerous fully developed plastoglobules (PGs), however, white-flowered individuals showed abnormal chromoplasts with few PGs (Fig. 2b,c).

### Preliminary mapping of the *Brwf* genes

To determine the locations of genes controlling white flower trait, 81 insertion/deletion (InDel) markers distributed on 10 chromosomes were developed based on the re-sequencing data of the two parents, and 34 InDel markers (W1-W11, W101-W288) exhibited polymorphism between 15S1040 and 92S105 (Supplementary Table S1). These polymorphic markers were used for bulk segregant analysis (BSA) of flower color trait. As a result, six markers (W101, W105, W107, W112, W114, and W116) on chromosome A01 and three markers (W1, W5, and W11) on chromosome A09 were linked with *Brwf* genes. Among them, W105 and W112 markers, W5 and W11 markers were randomly chosen to assay 30 milky yellow-flowered and 30 pale yellow-flowered plants from F_2_ population. The results showed that W105 and W112 markers were linked with the *Brwf1* gene controlling milky yellow flower and W5 and W11 markers were linked with the *Brwf2* gene controlling pale yellow flower, which indicated that *Brwf1* and *Brwf2* were located on chromosomes A01 and A09, respectively.

**Figure 3:**
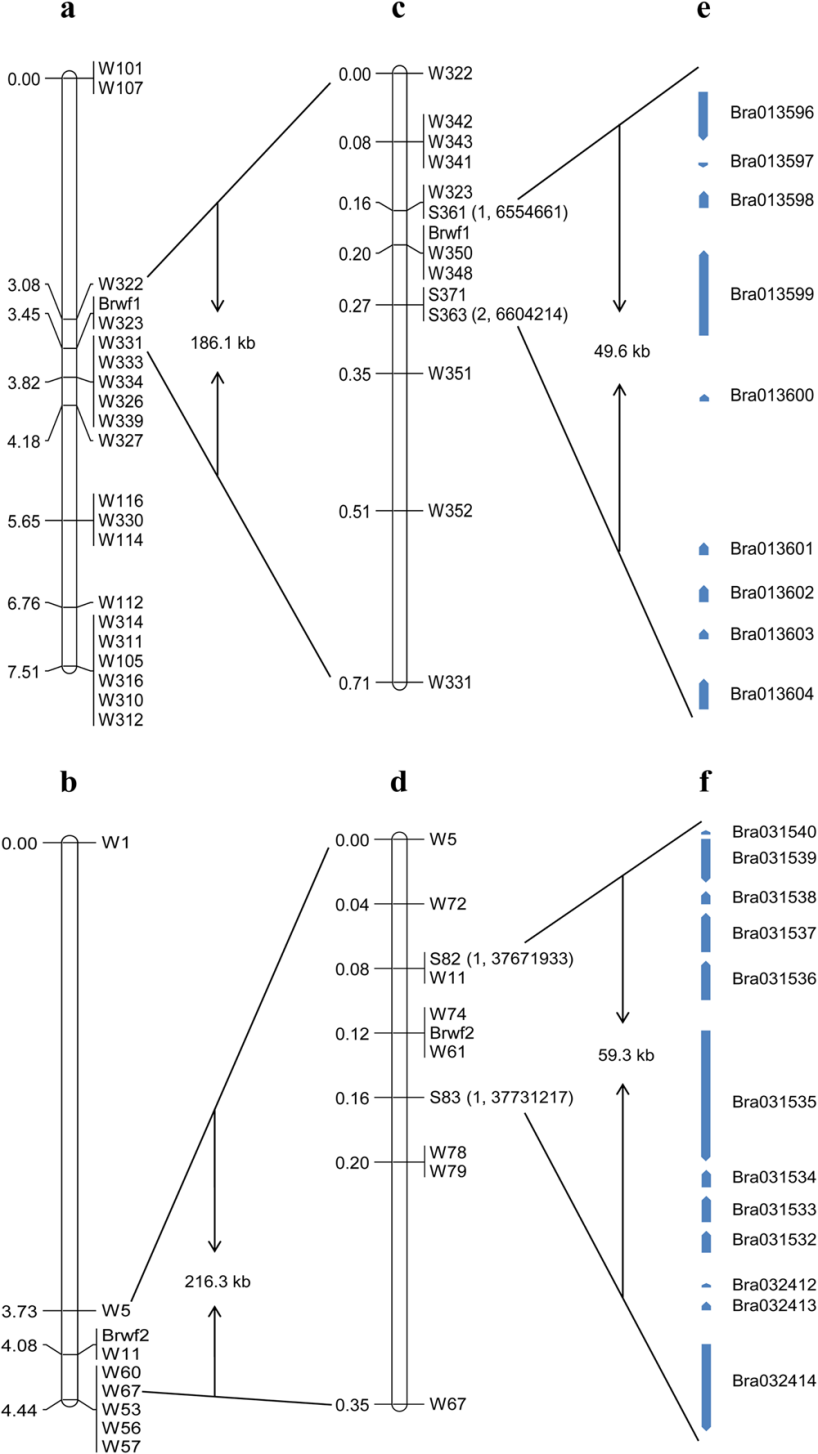
Fine mapping of *Brwf* genes in Chinese cabbage. Preliminary genetic map of *Brwf1* (**a**) and *Brwf2* (**b**) based on 144 F_2_ individuals. Fine linkage map of *Brwf1* (**c**) and *Brwf2* (**d**) based on 1282 F_2_ individuals. Annotated genes within the mapping intervals of *Brwf1* (**e**) and *Brwf2* (**f**). The bracket contains two numbers; the former represents recombinants between an individual marker and the white flower gene; the latter represents the physical position of SNP markers according to BRAD *B*. *rapa* reference genome.

For preliminary mapping of the *Brwf1* and *Brwf2* genes, newly designed 36 InDel markers on chromosome A01 and 35 InDel markers on chromosome A09 were screened between the two parental lines, and 14 (W310-W339) and 12 (W23-W60,W67) markers showed polymorphism, respectively (Supplementary Table S1). These polymorphic markers were used for BSA of flower color trait and 14 markers on chromosome A01 and 5 markers on chromosome A09 were linked with the *Brwf* genes. To preliminarily map the *Brwf1* and *Brwf2* genes separately, A and B groups that were segregated in *BrWF1*/*Brwf1* and *BrWF2*/*Brwf2* loci, respectively, were selected from F_2_ population and A group included 108 yellow-flowered and 36 milky yellow-flowered individuals and B group included 108 yellow-flowered and 36 pale yellow-flowered individuals. Then obtained 19 linkage markers from chromosomes A01 and A09 were used to detect A and B groups, respectively. In A group, the *Brwf1* gene, co-segregating with W323 marker, was localized to a region between W322 and W331 markers on chromosome A01, and the genetic and physical distances were 0.74 cM and 186.1 kb, respectively (Fig. 3a). In B group, the *Brwf2* gene was mapped to a 0.71 cM interval flanked by W5 and W67 markers with the corresponding physical distance of 216.3 kb on chromosome A09, and one marker W11 co-segregated with *Brwf2* (Fig. 3b).

### Fine mapping of the *Brwf* genes

**Figure 4:**
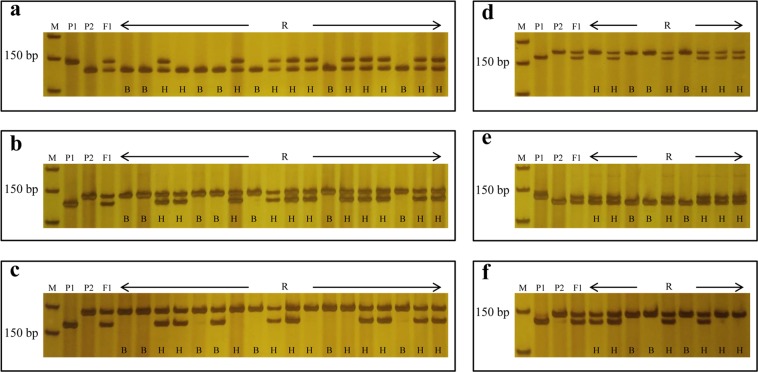
Genotyping of screened recombinants for fine mapping of *Brwf1* (**a-c**) and *Brwf2* (**d-f**) using closely linked markers. (**a**) W323, (**b**) W348, (**c**) W351; (**d**) W11, (**e**) W61, (**f**) W78. M: DL50 marker; P_1_: 92S105; P_2_: 15S1040; F_1_: F_1_ individual; R: screened recombinants from the F_2_ population using markers W322 and W331, W5 and W67; H: heterozygous genotype plant; B: recessive homozygous genotype plant.

For fine mapping of the *Brwf1* gene, the two markers W322 and W331 were used to detect recombination events in all F_2_ plants, and a total of 18 recombinants including 5 recombination events with W322 marker and 13 recombination events with W331 marker were obtained. Using the re-sequencing data of the two parents, 10 new InDel markers were developed from the preliminary mapping region and seven of them (W341-W352) exhibited polymorphism in the two parents (Supplementary Table S1). These polymorphic markers were unceasingly used to screen all the 18 recombinants. The results indicated that the *Brwf1* gene was delimited to a shortened interval between W323 and W351 markers with one recombinant and four recombinants, respectively (Fig. 4a–c and Supplementary Fig. S1). To further narrow down the mapping interval, two single-nucleotide polymorphism (SNP) markers (S361 and S371) were developed and used to test recombination events. As a result, one recombination event with S361 marker and two recombination events with S371 marker were found, and then a developed SNP marker S363 on the side of S371 was also used to detect two recombination events (Supplementary Table S1 and Fig. S2). The two SNP markers S361 and S363 further narrowed the *Brwf1* gene to an interval of 0.11 cM with the corresponding physical distance of 49.6 kb, Finally, two markers, W348 and W350, co-segregating with *Brwf1* were obtained (Fig. 3c).

To fine map the *Brwf2* gene, a total of nine recombinants were identified using W5 and W67 markers, which included three recombination events occurring between W5 marker and *Brwf2* and six recombination events occurring between W67 marker and *Brwf2*. Among 15 InDel markers developed from the preliminary mapping interval, five markers (W61, W72-W79) were polymorphic between 15S1040 and 92S105 (Supplementary Table S1). The nine recombinants were screened by five new polymorphic markers. As a result, the *Brwf2* gene was restricted to a region between W11 and W78 markers and there were one recombinant with W11 marker and two recombinants with W78 marker (Fig. 4d–f and Supplementary Fig. S1). For further narrow down the mapping region, two more SNP markers (S82 and S83) were developed for detecting recombination events (Supplementary Table S1). The result showed that one recombination event was found between each of the two SNP markers and *Brwf2* (Supplementary Fig. S2) respectively, so the *Brwf2* gene was delimited to a 0.08 cM region flanked by S82 and S83 markers, and the corresponding physical distance was 59.3 kb. Finally, two markers, W61 and W74, co-segregating with *Brwf2* were obtained (Fig. 3d).

### Identification and sequence analysis of the candidate genes

According to the *B*. *rapa* reference genome in BRAD (*Brassica* database, http://brassicadb.org/brad), 9 and 12 genes were annotated within the two final mapping intervals of *Brwf1* and *Brwf2* genes, respectively (Fig. 3e,f). Among 9 annotated genes in the *Brwf1* interval on chromosome A01, *Bra013602* encodes a plastid-lipid associated protein (PAP) that was previously reported to regulate carotenoid accumulation^39,40^ (Table 2). Out of 12 annotated genes in the *Brwf2* interval on chromosome A09, *Bra031539* was predicted to encode a carotenoid isomerase (CRTISO) that was involved in carotenoid biosynthesis^41–44^ (Table 2). Therefore, *Bra013602* and *Bra031539* were predicted as the two candidates for *Brwf1* and *Brwf2* genes, respectively.

**Table 2:** Annotated genes within the mapping intervals of *Brwf1* and *Brwf2* on chromosomes A01 and A09.

Chr.	*B*. *rapa*	Gene position^a^	Gene function^a^	*Arabidopsis thaliana* homolog
A01	Bra013596	6555476…6558338	Protein kinase	AT4G23280
	Bra013597	6559518…6559832	F-box family protein	AT4G22170
	Bra013598	6561855…6562856	Unknown protein	AT4G22190
	Bra013599	6567555…6572888	AKT2/3: cyclic nucleotide binding/inward rectifier potassium channel/protein binding	AT4G22200
	Bra013600	6577601…6578014	PRA1.H: prenylated PAB acceptor 1.H	AT4G27540
	Bra013601	6597194…6597946	ISU1: structural molecule	AT4G22220
	Bra013602	6598836…6599884	PAP: plastid-lipid associated protein	AT4G22240
	Bra013603	6600885…6601496	Zinc finger (C3HC4-type RING finger) family protein	AT4G22250
	Bra013604	6602225…6604058	Alternative oxidase	AT4G22260
A09	Bra031532	37708145…37710011	2-oxoglutarate-dependent dioxygenase	AT1G06650
	Bra031533	37705347…37707116	2-oxoglutarate-dependent dioxygenase	AT1G06650
	Bra031534	37703357…37704748	PSBP-1: Photo system II subunitp-1	AT1G06680
	Bra031535	37693586…37703026	Ribosome biogenesis	AT1G06720
	Bra031536	37686812…37689589	Unknown protein	AT1G06750
	Bra031537	37681637…37684412	GAUT6: Galacturonosyltransferase 6	AT1G06780
	Bra031538	37680010…37681151	RNA polymerase Rpb7 N-terminal domain-containing protein	AT1G06790
	Bra031539	37676627…37679733	Carotenoid isomerase	AT1G06820
	Bra031540	37674379…37674678	Glutaredoxin family protein	AT1G06830
	Bra032412	37717578…37717784	2-oxoglutarate-dependent dioxygenase	AT1G06640
	Bra032413	37718500…37719120	2-oxoglutarate-dependent dioxygenase	AT1G06650
	Bra032414	37722562…37728738	Unknown protein	AT1G06590

^a^Gene position and annotation based on BRAD *B*. *rapa* reference genome data (chromosome v1.5).

The specific primers WY503 was designed for cloning and sequencing of the cDNA sequences of *BrPAP* (Supplementary Table S1). The gene sequence comparison showed that there were 15 SNPs in the coding region of *BrPAP* between 92S105 and 15S1040 (Supplementary Fig. S3a), which resulted in four amino acid residue mutations (Supplementary Fig. S4a). Based on previous studies^42,44^, two designed primers, WY571 and WY572, were used to clone the cDNA sequences of *BrCRTISO* in 92S105 and 15S1040, respectively (Supplementary Table S1). The sequence alignment indicated that there were many SNPs, one small deletion, and one large insertion in the coding region of *BrCRTISO* in 15S1040. This large insertion had 943 bp that was located at the 3′ end of *BrCRTISO* (Supplementary Fig. S3b). After the amino acid sequence alignment, 17 amino acid residue changes and the deletion of two amino acid residues were found in BrCRTISO of 15S1040, however, at the 3′ end, the large insertion resulted in mutations of 15 amino acid residues, one amino acid residue insertion, and three amino acid residue deletions in BrCRTISO of 15S1040 (Supplementary Fig. S4b).

**Figure 5:**
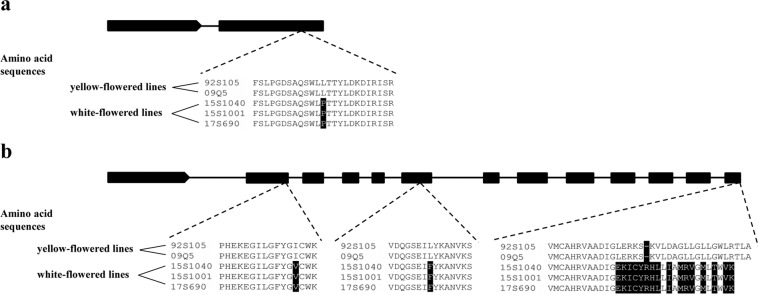
Gene structures and amino acid sequence analyses of BrPAP and BrCRTISO. (**a**) The coding region of *BrPAP* includes two exons and one intron. The nonsynonymous SNP mutation (T → C) in exon 2 results in the amino acid residue conversion (Leu → Pro) between yellow-flowered and white-flowered lines. (**b**) The coding region of *BrCRTISO* contains 13 exons and 12 introns. The nonsynonymous SNP mutation (A → G) in exon 2 and (C → T) in exon 6 and a large insertion in exon 13 cause the conversion of Ile to Val and Leu to Phe, and many amino acid residue changes, respectively, between yellow-flowered and white-flowered lines. The above amino acid residue mutations are consistent with flower color phenotypes. *Black backgrounds* indicate mutant amino acid residues. 92S105 and 09Q5 are yellow-flowered lines; 15S1040, 15S1001, and 17S690 are white-flowered lines.

To identify the key mutations of the two candidate genes between white-flowered and yellow-flowered lines, the genomic sequences of two candidate genes from one yellow-flowered line (09Q5) and two white-flowered lines (15S1001 and 17S690) were cloned using designed specific primers, which included WY503 for *BrPAP* of yellow-flowered and white-flowered lines, and WY561, WY562, WY563 for the *BrCRTISO* of yellow-flowered line and WY561, WY562, WY566 for the *BrCRTISO* of white-flowered lines according to previous studies^42,44^ (Supplementary Table S1). The deduced amino acid sequences of BrPAP and BrCRTISO from three yellow-/white-flowered lines were compared with that from the two parental lines. The results indicated that the deduced amino acid sequence of BrPAP in 09Q5 was same as that in 92S105, while there were seven amino acid residue mutations among 15S1040, 15S1001, and 17S690, but only one mutant amino acid residue (Leu → Pro) was found between two yellow-flowered and three white-flowered lines and it was located in the conserved domain of BrPAP (Fig. 5a; Supplementary Fig. S4a); the deduced amino acid sequence of BrCRTISO in 09Q5 had 17 amino acid residue mutations and one deletion of two amino acid residues compared with 92S105, while the sequences from 15S1001 and 17S690 were identical to that from 15S1040, however, two amino acid residue mutations (Ile → Val, Leu → Phe) and many amino acid residue changes at the end of sequences were consistent with the flower color and the two amino acid residues were located in the conserved domain of BrCRTISO (Fig. 5b; Supplementary Fig. S4b).

### Expression analysis of the candidate genes and carotenoid metabolic genes

**Figure 6:**
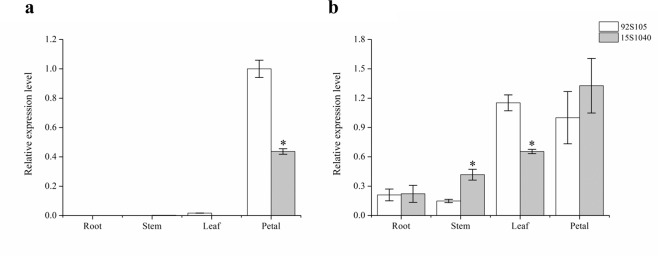
Expression pattern analysis of *BrPAP* (**a**) and *BrCRTISO* (**b**) in different tissues of 92S105 and 15S1040. Error bars represent the SD, and asterisks indicate significant difference (t-test, *P* < 0.05) between 92S105 and 15S1040. The expression of the two genes in petals of 92S105 was considered as the standard of ‘relative’ expression.

Expression pattern analysis of *BrPAP* and *BrCRTISO* was conducted using Quantitative real-time PCR (qPCR) in different tissues (roots, stems, cauline leaves, and petals) from the two parental lines. *BrPAP* expressed mainly in petals and could hardly be detected in other tissues with expression level of *BrPAP* in petals of 92S105 being twofold higher than that in 15S1040 (Fig. 6a); *BrCRTISO* had relatively higher expression levels in cauline leaves and petals than in roots and stems, however, *BrCRTISO* did not exhibit significant difference in expression between the petals of the two parental lines (Fig. 6b). Moreover, the expression levels of genes related to carotenoid metabolism in petals were detected. The results indicated that *CRTISO* and *Lycopene ε-cyclase* (*LCYE*) had no significant differences in expression between the petals of the two parental lines, but expression of other seven genes showed down-regulated in petals of 15S1040 compared with 92S105 (Supplementary Fig. S5).

## Discussion

In *Brassica* species, genetic analysis of flower color traits has been carried out early^16,17,36^. The previous investigations showed that white flower trait was dominant over yellow flower and controlled by a single gene in *B*. *napus*^11,45^ and *B*. *oleracea*^13,14,18,46,47^. However, several studies have reported that white flower trait was a recessive trait controlled by two major genes^20,37,38,48^. In this study, genetic analysis of white flower trait in *B*. *rapa* was conducted with F_2_ population derived from a cross between white-flowered line 15S1040 and yellow-flowered line 92S105. Our results showed that white flower trait was controlled by two separate loci and the white flower trait is recessive to yellow flower, consistent with previous reports^20,37,38,48^.

Multiple studies have reported recently on gene mapping of white flower trait in *Brassica* species. In *B*. *napus*, a white flower gene was mapped to a 0.39 cM region on chromosome C03^11^. Ashutosh *et al*.^46^ and Han *et al*.^12,47^ also mapped a white flower gene on chromosome C03 using populations derived from the crosses between broccoli and Chinese kale, cabbage and Chinese kale, respectively. In Chinese kale, a white flower gene was also delimited to chromosome C03^13,14^. The above results indicated that a single gene controlling white flower trait might be the same gene in *B*. *napus* and *B*. *oleracea*. In *B*. *juncea*, two recessive genes that controlled white flower trait were restricted to chromosomes A02 and B04 and the genetic distances were 0.13 cM and 0.25 cM, respectively^37,38^. In this study, we found that white flower trait in *B*. *rapa* was also controlled by two genes (*Brwf1* and *Brwf2*), which were mapped to intervals of 0.11 cM and 0.08 cM on chromosomes A01 and A09, respectively.

PAP, also called fibrillin, found in the pepper fruit chromoplasts and its homologous protein in chromoplasts of cucumber flower, was named as chromoplast-specific carotenoid-associated protein (CHRC)^49^. In chromoplast, *fibrillin* and *CHRC* were positively associated with carotenoid accumulation^39,40^. The suppression of the expression of *CHRC* gene in tomato flowers resulted in decreased carotenoids^39^, which indicated that *CHRC* plays a role in mediating carotenoid storage in chromoplasts of flowers. Over-expression of the pepper *fibrillin* gene in tomato increased the levels of carotenoids in fruit^40^. In this study, the *Bra013602* gene encoding PAP was located in the final mapping region of *Brwf1*, which deduced amino acid sequence has four amino acid residue mutations between the two parents and one of the mutations (Leu → Pro) occurred in the conserved domain of BrPAP between yellow-flowered lines (92S105 and 09Q5) and white-flowered lines (15S1040, 15S1001, and 17S690), which might affect the function of BrPAP in white-flowered lines. In addition, fibrillin was involved in plastoglobule formation based on previous investigations^40,50^. Over-expression of the *fibrillin* gene from pepper in tobacco resulted in the increased number of PGs in plastids of leaves and petals^50^. In this study, ultrastructural analysis of chromoplasts in the two parents revealed that the number of PGs in yellow petal chromoplasts was more than that in white petal chromoplasts. Expression pattern analysis of *BrPAP* indicated that the expression level of *BrPAP* in petals was much higher than that in other tissues. These results indicated that *BrPAP* was the most possible candidate for white flower trait.

It was known that the role of CRTISO is the control of the conversion of prolycopene to lycopene. The functional disruption of *BrCRTISO* gene resulted in the orange head leaf formation in Chinese cabbage^41–44^. However, Lee *et al*.^22^ reported that 19 amino acid residue changes and deletion of two amino acid residues were found in the amino acid sequence of BrCRTISO from pale-yellow flower cultivar compared with that in yellow flower cultivar. In this study, *Bra031539* encoding BrCRTISO was located in the final delimited genomic region of *Brwf2*. The amino acid sequence analysis of BrCRTISO indicated that two amino acid residue mutations (Ile → Val, Leu → Phe) that were located in the conserved domain of BrCRTISO and many amino acid residue changes at the end of sequences were found between two yellow-flowered lines (92S105 and 09Q5) and three white-flowered lines (15S1040, 15S1001, and 17S690). In addition, although Zhang *et al*.^42^ reported that the amino acid residue mutation (Leu → Phe) of BrCRTISO could not affect the protein function in leaves, this mutation which was found in petals might affect BrCRTISO function in this study. Taken together, two amino acid residue mutations (Ile → Val, Leu → Phe) and many amino acid residue changes in the C-terminal end of BrCRTISO might affect its function, which suggested that *BrCRTISO* was the most promising candidate for white flower trait.

**Figure 7:**
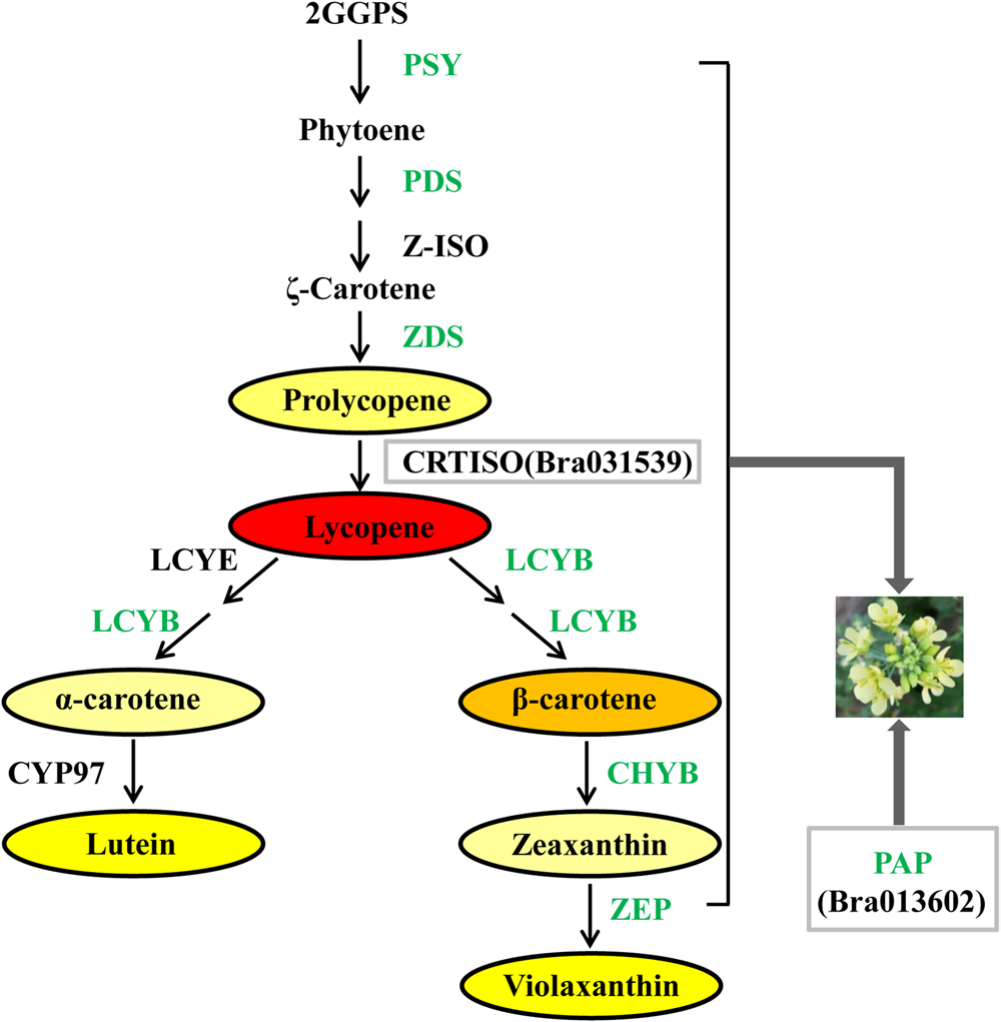
Proposed molecular mechanism diagram of white flower formation in Chinese cabbage. PSY: phytoene synthase, PDS: phytoene desaturase, Z-ISO: ζ-carotene isomerase, ZDS: ζ-Carotene desaturase, CRTISO: carotenoid isomerase, LCYE: Lycopene ε-cyclase, LCYB: Lycopene β-cyclase, CHYB: β-carotene hydroxylase, CYP97: cytochrome P450-type monooxygenase 97, ZEP: zeaxanthin epoxidase, PAP: plastid-lipid associated protein. Enzymes with green represent the genes that encode these enzymes were down-regulated expression in white flower. Gray frames represent mutated genes.

In *B*. *napus*^11^ and *B*. *juncea*^37^, the major carotenoid in yellow and white petals was violaxanthin, but the total carotenoid contents in yellow petals were forty-twofold and eightfold higher than that in white petals, respectively. In the present study, carotenoid analysis of yellow and white petals showed that violaxanthin and lutein were mainly accumulated in yellow and white petals of Chinese cabbage, however, the total carotenoid content was twenty times higher in yellow petals than in white petals, which were consistent with the previous studies^11,37^. Moreover, because light could partially replace CRTISO activity^22,44^, which combined with the phenotypic observation of F_2_ plants and the results of amino acid sequence comparison of BrPAP and BrCRTISO, we hypothesized that the mutations of *BrPAP* and *BrCRTISO* and light might jointly affect the prolycopene accumulation and resulted in barely detecting it in 15S1040. In *B*. *napus*, a single dominant gene, *BnaCCD4*, controls the white flower trait and associated with carotenoid degradation^11^. In *B*. *juncea*, the white flower trait was jointly controlled by two recessive genes, *Bjpc1* and *Bjpc2* which encode esterase/lipase/thioesterase family protein and phytyl ester synthase 2, respectively, and were involved in carotenoid esterification^37,38^. In this study, the potential candidate genes for the white flower trait in Chinese cabbage were *BrPAP* and *BrCRTISO* that were associated with carotenoid storage and biosynthesis, respectively. The results of TEM analysis and amino acid sequence alignment of BrPAP indicated that the mutation of *BrPAP* resulted in decrease of carotenoid accumulation by blocking PG formation. The mutant types of BrCRTISO in the present study were incompletely consistent with the previous investigations^42,44^, which indicated that the function of BrCRTISO in 15S1040 might not be complete disruption. In addition, expression analysis of genes associated with carotenoid metabolism showed that the majority of carotenoid biosynthesis pathway genes were down-regulated expression in petals of 15S1040 compared with 92S105. Hence, the mutation of *BrCRTISO* might decrease the flux of carotenoid biosynthesis pathway. Taken together, we inferred that both mutations of *BrPAP* and *BrCRTISO* maybe lead to the white flower formation by decreasing total carotenoid content in 15S1040 (Fig. 7).

## Methods

### Plant materials

The five Chinese cabbage lines, the white-flowered 15S1040, 15S1001, 17S690, and the yellow-flowered 92S105 and 09Q5, were used in this study. 15S1040 and 92S105 (Fig. 1a,b) were selected as parents for constructing F_2_ population. To study the inheritance pattern of white flower trait and fine map the *Brwf* genes, a cross between the two parental lines, 15S1040 and 92S105, was used to produce the hybrid F_1_, then one F_1_ plant was self-pollinated to generate the F_2_ population with 1282 individuals. Other white-flowered and yellow-flowered lines were used for the identification of the candidate genes. All materials were bred and provided by the Chinese cabbage research group at the Northwest A&F University, Yangling, China.

All plants used in the present study were grown and naturally vernalized at the experimental field of the Northwest A&F University in 2018. During the flowering stage, the observations of at least ten flowers per plant were performed twice to evaluate the flower color of each individual with an 8-day interval.

### Carotenoid extraction and analysis

Carotenoid were extracted from fresh petals at the flowering stage and detected following the methods of Cao *et al*.^51^. Carotenoid analysis was performed using LC-2010AHT HPLC (Shimadzu, Kyoto, Japan) with C30 column (YMC, Kyoto, Japan). Carotenoids were identified by the typical retention time of the standard compounds, including violaxanthin (Sigma-Aldrich, Saint Louis, America), lutein (Solarbio, Beijing, China), α-carotene and β-carotene (Wako, Osaka, Japan). The identification of prolycopene was performed based on reported the typical retention time and relative order of carotenoid compound peaks^22,43,51^. Carotenoid content was quantified according to Morris’ method^52^. The total carotenoid content was the sum of all the detected carotenoid compound contents. Three biological replicates were used for all analyses and the calculation of means and standard deviations were conducted. The significant difference between 92S105 and 15S1040 was analyzed by t-test.

### Transmission electron microscopy analysis

Petals from 92S105 and 15S1040 flowers at the flowering stage were cut into 0.3 × 0.6 cm sections, fixed with 2.5% glutaraldehyde. The preparation of observation samples of petals and TEM analysis were performed according to Yi *et al*.^53^ described methods.

### DNA and RNA extraction, first-strand cDNA synthesis, and gel electrophoresis

Total genomic DNA was isolated from fresh leaves using the cetyl trimethylammonium bromide (CTAB) method described by Porebski *et al*.^54^. Using the MiniBEST Plant RNA Extraction Kit (TaKaRa, Dalian, China), total RNA was extracted from petals of open flowers, roots, stems, and cauline leaves from the two parental lines, and first-strand cDNA, which was used for quantitative real-time PCR (qPCR), was synthesized by PRIMESCRIPT 1st Strand cDNA Synthesis Kit (TaKaRa, Dalian, China). To clone the cDNA sequences of the candidate genes, first-strand cDNA synthesis was performed with PRIMESCRIPT II 1st Strand cDNA Synthesis Kit (TaKaRa, Dalian, China).

Two (yellow and white flowers) and four (yellow, milky yellow, pale yellow, and white flowers) kinds of F_2_ individuals were used for BSA and fine mapping, respectively. Two DNA pools, yellow-flowered pool and white-flowered pool, were created by mixing equal amounts of DNA from 8 individuals with yellow flower and 8 individuals with white flower, respectively, which were randomly selected from F_2_ population. The PCR reaction and separation of its products were performed as described by Zhang *et al*.^31^.

### Development of InDel and SNP markers

To develop InDel and SNP markers, the two parental lines, 15S1040 and 92S105, were re-sequenced with HiSeq X Ten (Gene Denovo, Guangzhou, China) at 30- and 91-fold sequencing depths. The re-sequencing data of 15S1040 and 92S105 were mapped to the *B*. *rapa* reference genome in BRAD, the genomic variants were found using Genome Analysis Toolkit (GATK), and the annotation of the physical location of each genomic variant was carried out. The insertions/deletions> 3 bp and single-nucleotide polymorphism loci were used to develop InDel and SNP markers, respectively, with the Primer Premier 5.0 (http://www.premierbiosoft.com/primerdesign/) software based on the corresponding flanking sequences in the *B*. *rapa* reference genome. The primers used in the present study were synthesized by Sangon Biotech Co., Ltd (Shanghai, China).

### Identification of recombination events

To obtain the DNA fragments that contained SNP loci in the recombinants, the specific primers were designed according to the reference genome of *B*. *rapa*. The purification of PCR products and sequencing were conducted using our previous method^55^. The nucleotide sequences were analyzed using the DNASTAR Lasergene 7.1 (http://www.dnastar.com) and Chromas 2.4.1 (http://technelysium.com.au/wp/chromas/) softwares.

### Fine mapping of the *Brwf* genes and identification of the candidate genes

The polymorphic molecular markers were utilized to assay genotype of plants in the F_2_ populations. The linkage analyses were conducted using the genotypic data of the polymorphic markers and phenotypic data of each individual in F_2_ segregating population. The linkage map was then constructed using the JoinMap 4.0 (https://www.kyazma.nl/index.php/JoinMap/) software based on a LOD threshold score of 6.0. The candidate genes in the final delimited region were analyzed based on the annotation data of the *B*. *rapa* reference genome in BRAD.

### Cloning and sequence analysis of the candidate genes

To clone the DNA and cDNA sequences of the putative candidate genes, the primers were designed according to the *B*. *rapa* reference genome. The cloning of putative candidate genes and sequencing were performed according to our previous method^55^. The complete coding sequences of two candidate genes from two yellow-flowered and three white-flowered lines were submitted to GenBank, the accession numbers: *BrPAP*: MN338556 (92S105), MN338557 (09Q5), MN338558 (15S1040), MN338559 (15S1001), and MN338560 (17S690); *BrCRTISO*: MN338561 (92S105), MN338562 (09Q5), MN338563 (15S1040), MN338564 (15S1001), and MN338565 (17S690).

### Expression analysis of the candidate genes and carotenoid metabolic genes

qPCR was used to investigate the expression pattern of the candidate genes in different tissues and the expression levels of carotenoid metabolic genes in petals of the two parental lines, and carotenoid metabolic genes included *phytoene synthase* (*PSY*), *phytoene desaturase* (*PDS*), *ζ-Carotene desaturase* (*ZDS*), *carotenoid isomerase* (*CRTISO*), *Lycopene ε-cyclase* (*LCYE*), *Lycopene β-cyclase* (*LCYB*), *β-carotene hydroxylase* (*CHYB*), *zeaxanthin epoxidase* (*ZEP*), and *carotenoid cleavage dioxygenases 4* (*CCD4*). The specific primers were designed for qPCR using the Primer Premier 5.0 software (Supplementary Table S2), and Chinese cabbage *elongation-factor 1α* (*EF1α*) gene was selected as the internal reference^56^. The qPCR tests were performed following the method described by Ren *et al*.^57^. All gene expression analyses were repeated three times with independent samples. The calculation of relative expression level was performed using the 2^−ΔΔCT^ method^58^. The significant difference analysis of expression data between 92S105 and 15S1040 was performed using t-test.

## Supplementary information

Supplementary Information.
