# Supplementary material for: Fine mapping and candidate gene analysis of the white flower gene Brwf in Chinese cabbage (Brassica rapa L.)
Source: Sci Rep. 2020 Apr 8;10:6080. doi: 10.1038/s41598-020-63165-7 (PMC7142070; doi:10.1038/s41598-020-63165-7)
Supplement: Supplementary file 1 — Supplementary Information. [file 41598_2020_63165_MOESM1_ESM.pdf]

Fine mapping and candidate gene analysis of the white flower gene *Brwf* in Chinese cabbage (*Brassica rapa* L.)

Ning Zhang[1], Lin Chen[1], Shuai Ma[1], Ruofan Wang[1], Qiong He[1], Min Tian[1] & Lugang Zhang[1*,2]

[1] State Key Laboratory of Crop Stress Biology for Arid Areas, College of Horticulture, Northwest A&F University, Yangling 712100, Shaanxi, China

[2] State Key Laboratory of Vegetable Germplasm Innovation, Tianjin 300384, China

***Correspondence author**

Lugang Zhang

State Key Laboratory of Crop Stress Biology for Arid Areas, College of Horticulture, Northwest A&F University, Yangling 712100, Shaanxi, China

E-mail: lugangzh@163.com

Supplementary Table S1. The primer sequences used for gene mapping and cloning of the candidate genes in this study.

Marker	Type	Chr.	Position	Primer sequence (5' to 3')	
				**Forward primer**	**Reverse primer**
W1	InDel	A9	37545087	TCATTAGGCATTGTTTGGC	CTTCAACGCTTCATGGATG
W5	InDel	A9	37567264	TATCCTCATGATCACAGCAG	GGAAGAACCAAGACATTGAA
W11	InDel	A9	37663645	TCATGTTGCTAACCGACTG	TTCATACCAAACACGACCA
W23	InDel	A9	25357859	AAGGTTCGCTGTAAAGGTG	AGTAACTGTTTGGATATTGCC
W24	InDel	A9	25727342	CAAGCTACCGATTTCTTATATC	AGAGCCTTAAACCGTGTTG
W25	InDel	A9	26626596	CGGTTCTGAGGTCTCACA	GCCTCTCTACCTTCAGCTT
W28	InDel	A9	27981750	GAAGCAGGCTCATAACTATTTC	GCCTTTAATCTCTTCCCTC
W29	InDel	A9	28603636	TCACTTTGTGCTGCTCTTG	GGTTTTGATGGCATAGATG
W35	InDel	A9	31796694	GTGAAGTTGAAAGGCCATA	GTCAGCTTTTTGACCAATTC
W39	InDel	A9	34388774	GTATGATCTTGGATTGTGCC	GTACCGAGAATAAACAAGGC
W53	InDel	A9	37789592	ATAAGTTCAAATTCGCCCC	CCGTGTTCTAAAAATGCCT
W56	InDel	A9	38168007	GTAACTATGCAACGAAACCG	ATCATACTTCAGCAGACCG
W57	InDel	A9	38267460	GATCTACACTCTCGATCTCCA	TTTCGTGGTCCTTGTTCAG
W60	InDel	A9	38751858	CATCCCCATCCTAACTTCA	ACCATAACCAATACGCTGC
W61	InDel	A9	37678370	CCCATATAAAGCTTCCAACTAC	GAACTCTCTTCCATCTGCTAG
W67	InDel	A9	37783554	CATCACACTGCTTTGTTCAC	GCTTGGGAGTTCGTGTATT
W72	InDel	A9	37654068	TGCCCTTTTATAGCATGAGC	TGTGGATCTGTGAGCAAATG
W74	InDel	A9	37684484	CTGGAGATGTCCTGCTTGA	ACTCCCCCAGAAACTGAAG
W78	InDel	A9	37733133	CTCTCATCTTCGTGTTCCTC	GAAAAGGGATGGGATTGAG
W79	InDel	A9	37745289	TGGCTGAGGAAGATTTTGC	CCTGAGGTTGTGTGTGCAT
W101	InDel	A1	4069774	CCACCTACACCTCCTAAACC	GCCTTGATGACCAAAAGAAC
W105	InDel	A1	19835745	ATCCCAAACACGAAAGCC	ATGTGGAGGTCTCTGGAAA
W107	InDel	A1	5489792	TGAGTCTCTCATTGACCGTG	GCACATCATTAGGAGTGTGAC
W112	InDel	A1	10737370	GCAAAAATGAAATCAGAGAGC	ACAAGGGTCAATATGCTGTC
W114	InDel	A1	9199516	GGATAGATGTGTGTTTGGTC	TAGGAATGTTGAGCCCTTAA
W116	InDel	A1	8412407	GAGCAGCAAGTAAAGAAGC	TCGGTGGAGATACCAAAT
W121	InDel	A2	3068227	AACTCTGCTCATGCCAAAA	TTGCCTCGTCTCTTCTTG
W126	InDel	A2	23891459	GGCTGCATTGTCAGGTTTA	CGTCTTCTTTCAACTGTGGA
W144	InDel	A3	16218372	TATCTTGCTTTTGGCTGGTC	GGAACAATTAAGCTGCACAT
W145	InDel	A3	20688416	ACCAAACTGAGACCAAATGA	TGAAGGTGACAGAATCAAGAG
W161	InDel	A4	2096628	TATTTTCTGCCATATTCACC	ACATTGATTTCTGGTGCAT
W163	InDel	A4	6794698	ACATTGATTTCTGGTGCAT	AAGCAAAGATCCTTCCACT
W165	InDel	A4	11192839	GTTTCAGCGGAAGAATATCC	GAATCTTTGACACAATGACCAG
W167	InDel	A4	15350417	TTAAACCCCATTTTACCTGC	TGTACAAGTGGCAGCAAGTC
W168	InDel	A4	16702011	TGCCACAAGATTTTCTTGAC	TGTTAGGATTCTGAGCACAA
W183	InDel	A5	12353202	AGAGAGTGGTAGCAGCCGTA	CTCGCTCCATCCTAGTTGT
W186	InDel	A5	15135858	CTTTTCAAACAACACATGTACCT	TTCAATAACGAACACTTGCG
W202	InDel	A6	7713213	GTGGATGGATGAATGAGGAG	TTCGTTCTGACTCTACGTGC
W204	InDel	A6	16994461	AATAGTCGAATGAATGATGC	TTTTGTACGCAGGGCTTTTA
W222	InDel	A7	10266722	ATCTCCCTCCAAACCTTCTA	GCTTCGTAAGATTGGCATG
W224	InDel	A7	20566080	CGGAACACCAGCAGTATAG	CTTTTGCTGCAGTGAAAAAC
W244	InDel	A8	10931466	AACTCCAATGGGTAGAACC	ATTCAGCTCTTGATGCTGCT
W247	InDel	A8	19041501	TATGGAGGTTGGTTCCACA	GTTACAATGGCTGCCATGT
W264	InDel	A9	15123782	CCCATTTCCTTGCCCTATA	AAGACGGGACATTTTGCTG
W265	InDel	A9	10927805	GTTATTTTGTCCGTGCCAC	AAAGTGAGTTGCGTCTTCG
W268	InDel	A9	16348066	AGGCAAAACAACACTAACG	AGCATTGTGGGTTGACTTT
W269	InDel	A9	16302887	CAATGCCATTCCATCTTCAG	GGCTGTCTTCTTCCAGGTA
W282	InDel	A10	3375428	CATCGCAGCAGTCCTTATC	GGGGCCAAGAAACTAGTCT
W283	InDel	A10	8019503	CAAAGAAGGGCCATGTAAGA	GACTAGGGATGGGATCAAAC
W287	InDel	A10	13224240	ACGTGTCTTCGCTTGTAGT	CAACTTGCTTTCACATTGA
W288	InDel	A10	15732241	CATTCGATCCTTTGTTTTACTTC	GATAATGCTTTGGTTGTTGC
W310	InDel	A1	16816608	TATAGGCTTGCGAGTAAATG	GGATAGTTTGGAATTTCCAG
W311	InDel	A1	16855441	AGAACATAGTGCCACGAATG	GCCATTTGCAGCTAATAAAC
W312	InDel	A1	17396304	GCTTAAAAAGAACTCATCTC	TTCAGGAAGAAATCTATGGG
W314	InDel	A1	18248879	TCTATAATCCTGTGGCGTACT	CACGTCCACATATAAAAGCTG
W316	InDel	A1	18636471	CATAGGGTTTCATAACTGGG	GGTGCTCATGTTCTTGAGTT
W322	InDel	A1	6461743	GAGAGAGCCTTTCCTTATTTC	AAGCTGCATTTAATGGGAC
W323	InDel	A1	6553957	ATCATTATTGCCCCTACCG	CAGCGATCTTCTCAGTTGT
W326	InDel	A1	7377149	GCCATCAATAGACGAAGAG	ATCTTAGTCTTGGTGGGAAC

Marker	Type	Chr.	Position	Primer sequence(5' to 3')	
				**Forward primer**	**Reverse primer**
W327	InDel	A1	7694766	CTTGATCTCTCTTGTTATCATCC	GTTGTTGCTTGCGTTTGAA
W330	InDel	A1	8344804	CCAGGATTGAGTACACCTG	GTAAAGATGAAACTCTGCGG
W331	InDel	A1	6647881	CAGATCAGTCACCTCCTCC	CAGAGAATTGAGACGGATGG
W333	InDel	A1	6824993	GTCCCACATAGCCCATTATA	GAAGCCATTGAGACTCACA
W334	InDel	A1	6877351	ACCTAACAATGAAGCAAGCC	CATGCACATGGCATTTCTT
W339	InDel	A1	7182971	GCGAGCACACTGTTCAAAA	CCATATTTTTCCTCTCGGG
W341	InDel	A1	6465617	GCTGATGCTAGAAGCCAAG	ATCGTACCAGTTAATCCGG
W342	InDel	A1	6491165	AACTCAAACAGGACGTCTAG	CACGATTATTGGACCACC
W343	InDel	A1	6509888	CCAACTCCGAGTCAAGAATC	GAACTCTTGAAGACGTTAGCG
W348	InDel	A1	6600745	GACTTAATGGGCCTTCTGA	GAAACCAATCGAATGGCTG
W350	InDel	A1	6602900	TGAAAAGTGATCTGCGTCG	ATCGTTAGCCCAAGAATGG
W351	InDel	A1	6636381	CGGTGAGCAAGCACTATAC	CCGAAAACAAGGCTACTGAC
W352	InDel	A1	6642716	TTTTCCTTGTGCATGTTGC	ACTCTATGGTCATGTAGTAACG
S82	SNP	A9	37671933	TGTCTTTGTCAATAGGCAG	AACAAGCTCACCTTCGTAA
S83	SNP	A9	37731217	CGTGTACTCTTGGATGTCC	CGTAATCTAGCGGAAACGA
S361	SNP	A1	6554661	TGCAAGATATCACGCATCAC	CGATCTTGTAAATGTAAGACGC
S363	SNP	A1	6604214	GAGGAGATGCCTGAAATCG	CCGATGAATGAATTGAGGTC
S371	SNP	A1	6627618	AGAACATTGTTGGCAAAGGG	GCTCGATCAGTAGTGACTTG
WY503		A1	6599884	ATGGCGACGGTTCAGTTCTTC	TCAAGGGTTCAAGAGAGGGCT
WY561		A9	37676607	GACGAACCAAGCTCAGAACCATG	CTTACAGCCTTTCCATCATCAA
WY562		A9	37677503	TCTGCTGGAAGGTTCTGATGT	GCTGAGGAAGATACTGCCATAA
WY563		A9	37678766	GCCATCATTTCGTACTTGAGG	CTTTGTGTCCTGTACCCTTTAGAG
WY566		A9	37678919	CCTCAGCATCCCAACCATTC	TCACAGAAACCAAAACGAGTTTTC
WY571		A9	37676627	ATGAATCTCTGTCTCCACAATCCC	CTATGCGAGTGTCCTTAACC
WY572		A9	37676627	ATGAATCTCTGTCTCCACAATCCC	TCACAGAAACCAAAAACGAG

Supplementary Table S2. The primer sequences used for qPCR examinations in this study.

Primer	Primer sequence (5' to 3')		Note
	**Forward primer**	**Reverse primer**	
EF1α	ATACCAGGCTTGAGCATACCG	GCCAAAGAGGCCATCAGACAA	Reference gene
PSY	CCAGACCCAATGAACACTTGTG	AAACCTTCTCTTCGGAGGAGAG	*Bra006391*
PDS	ACCTCTGTTGCTTGAAGCGA	TGGGAGGACATCTGGGAAAT	*Bra010751*
ZDS	ACTCCTGCTACTGCGTTTCTCT	ATCGAGAAGCTCAACAGCAGTC	*Bra040411*
LCYB	GAATCTCGACTTGGACCTTCCT	TGGTGTCTAGGCAGTCTAGCAA	*Bra029825*
LCYE	CACGACACTACTTCCTTCACCA	GCAAACAGAACCAGATCTCCTG	*Bra006838*
CHYB	CACCGTAACATTCAACCCTCTC	AACCTCTCGGACTTCTTCCTCT	*Bra019145*
ZEP	TGGGTTGTTCAACTCCCTTC	GGTTACTGTCTCCCGCTTCT	*Bra012127*
CCD4	TCGACTCGACCGGGAATAA	GAGCGATGAGAACGATGGTG	*Bra013378*
CRTISO	CTCCGCCTTGAAGACTTCAAA	CTTTAACAGCTAGCTGAGTCGC	*Bra031539*
PAP	CGCCGTTTCTCGTTCTGATT	TCAAACCTCGGTCTGTGCC	*Bra013602*

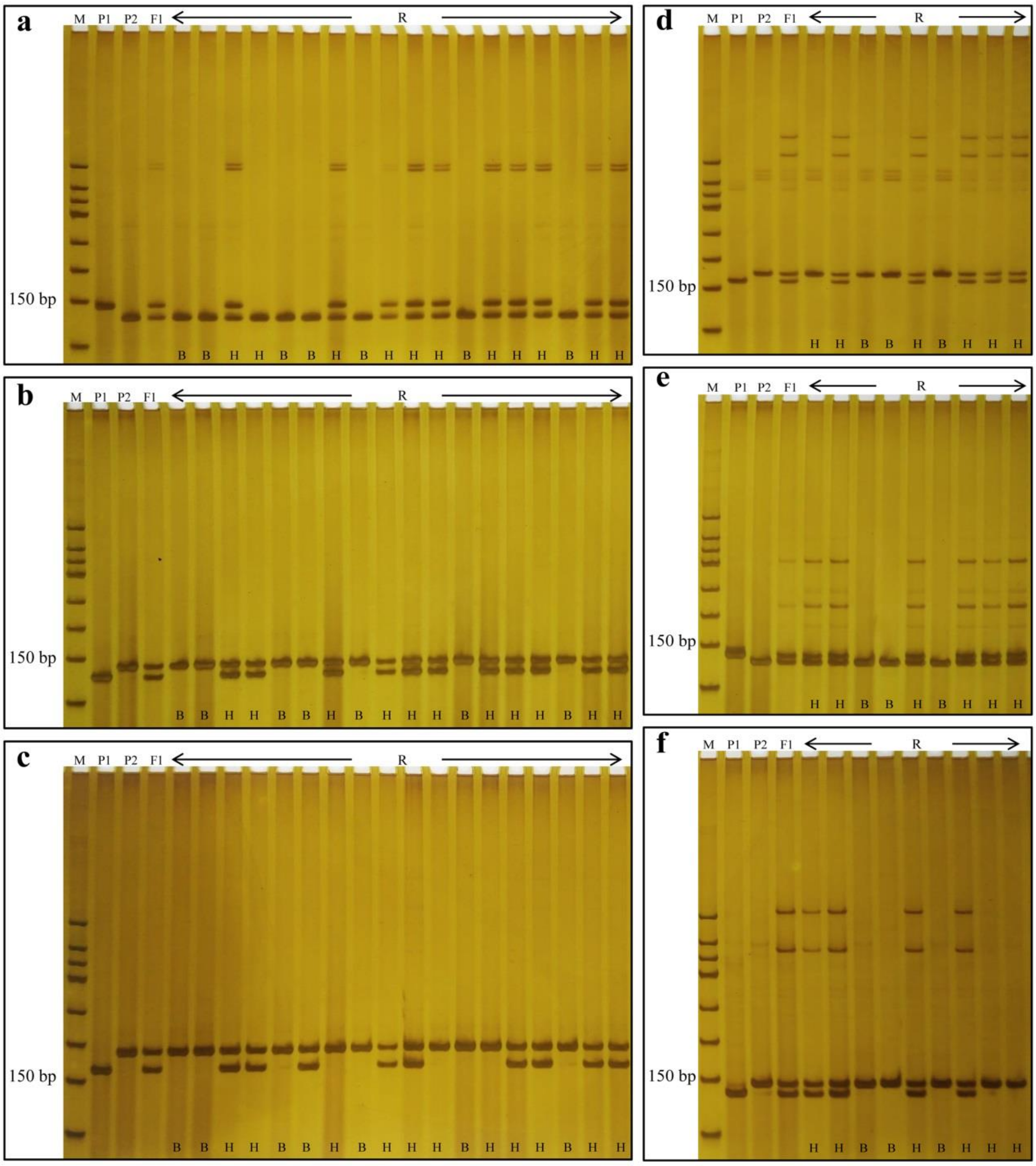

Supplementary Figure S1. Full-length images of gels exhibited in the Figure 4. (**a**) Fig. 4a, (**b**) Fig. 4b, (**c**) Fig. 4c, (**d**) Fig. 4d, (**e**) Fig. 4e, (**f**) Fig. 4f.

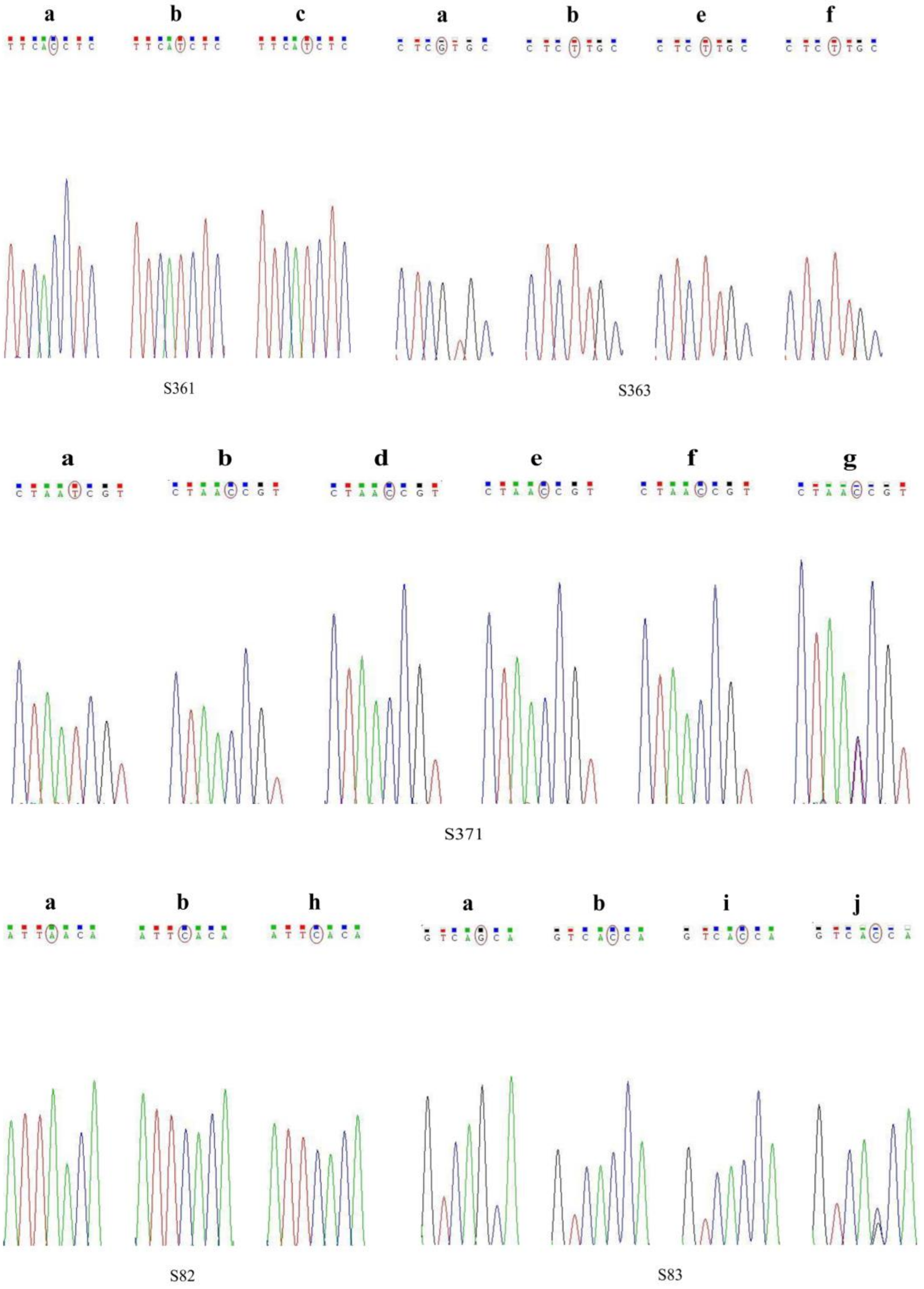

Supplementary Figure S2. Identification of recombination events of individuals (**c**-**j**) from F_2 population using SNP markers. The *red ellipses* represent the SNP loci. (**a**) 92S105, (**b**) 15S1040, (**c**), (**e**), (**f**), (**g**), (**h**), (**i**), (**j**) heterozygous genotype individual, (**d**) recessive homozygous genotype individual.

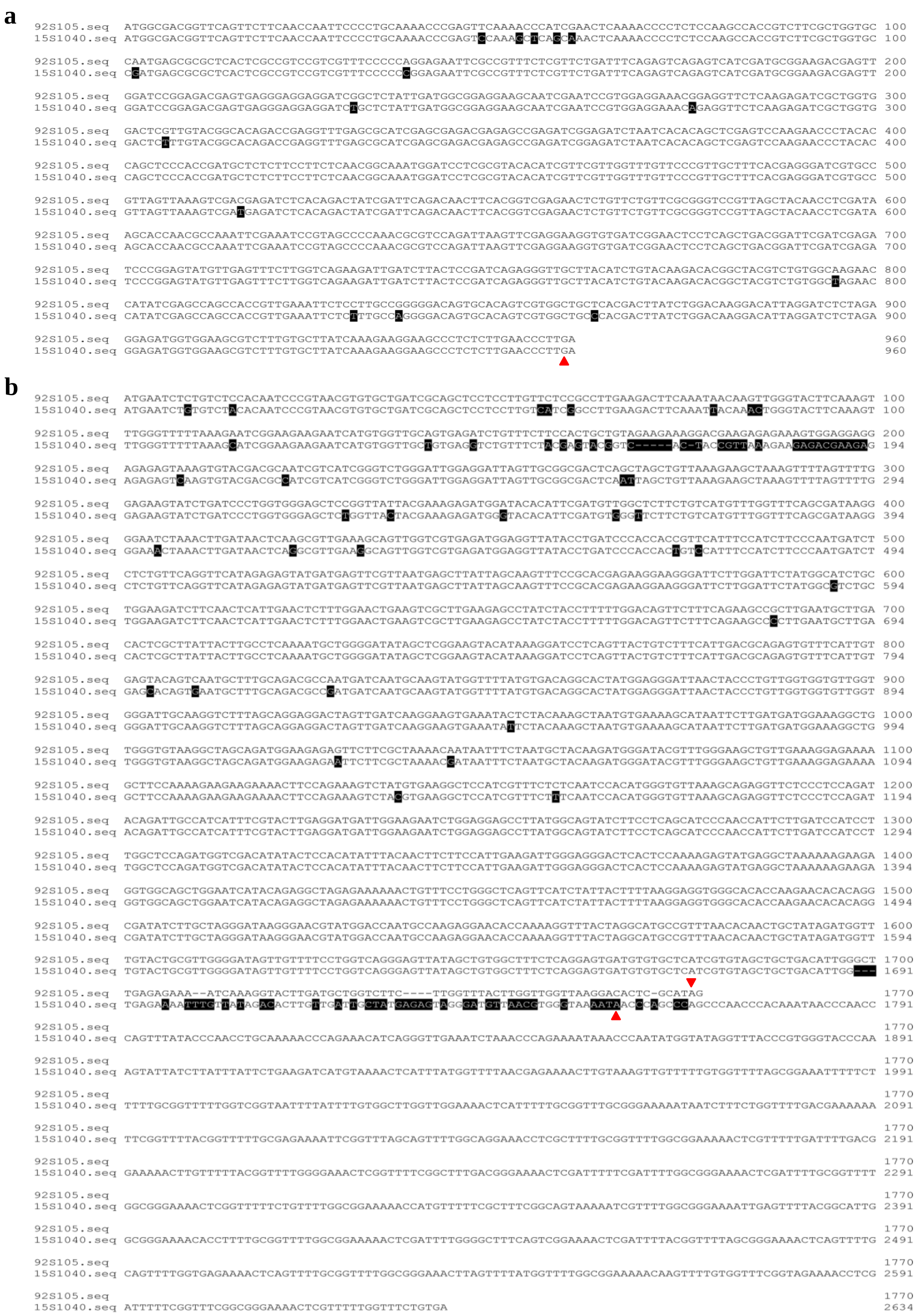

Supplementary Figure S3. cDNA sequence alignment of *BrPAP* (**a**) and *BrCRTISO* (**b**) between 92S105 and 15S1040. The mutations are indicated in *black background*. *Red triangles* indicate stop codons.

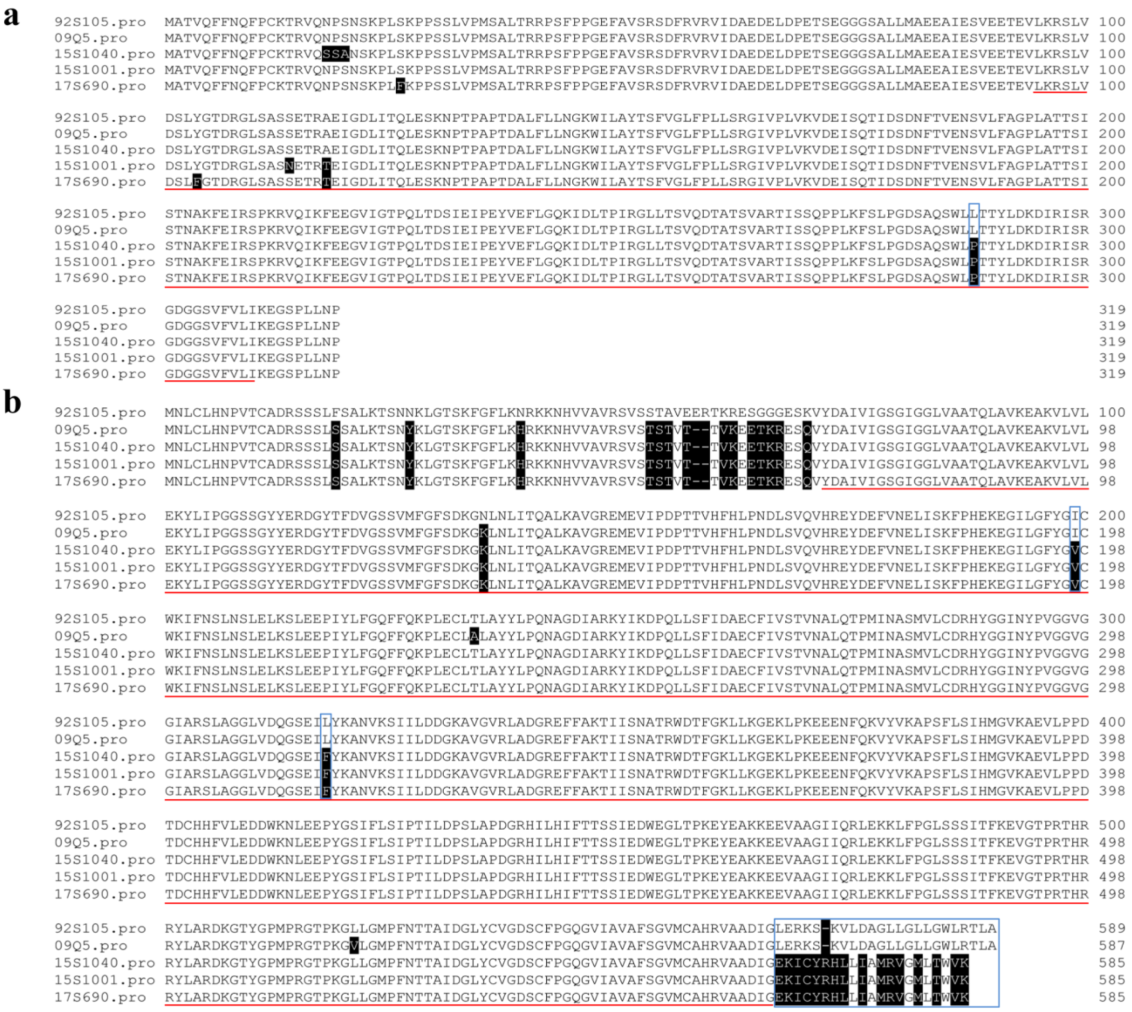

Supplementary Figure S4. Alignment of deduced amino acid sequences of BrPAP (**a**) and BrCRTISO (**b**) between yellow-flowered lines (92S105 and 09Q5) and white-flowered lines (15S1040, 15S1001, and 17S690). The *black backgrounds* indicate mutant amino acid residues. The *red lines* under the amino acid sequences indicate the predicted PAP and rossmann-fold NADP+ binding domains of BrPAP and BrCRTISO, respectively, based on NCBI annotated information. *Blue rectangles* represent the critical amino acid residue mutations between yellow-flowered and white-flowered lines.

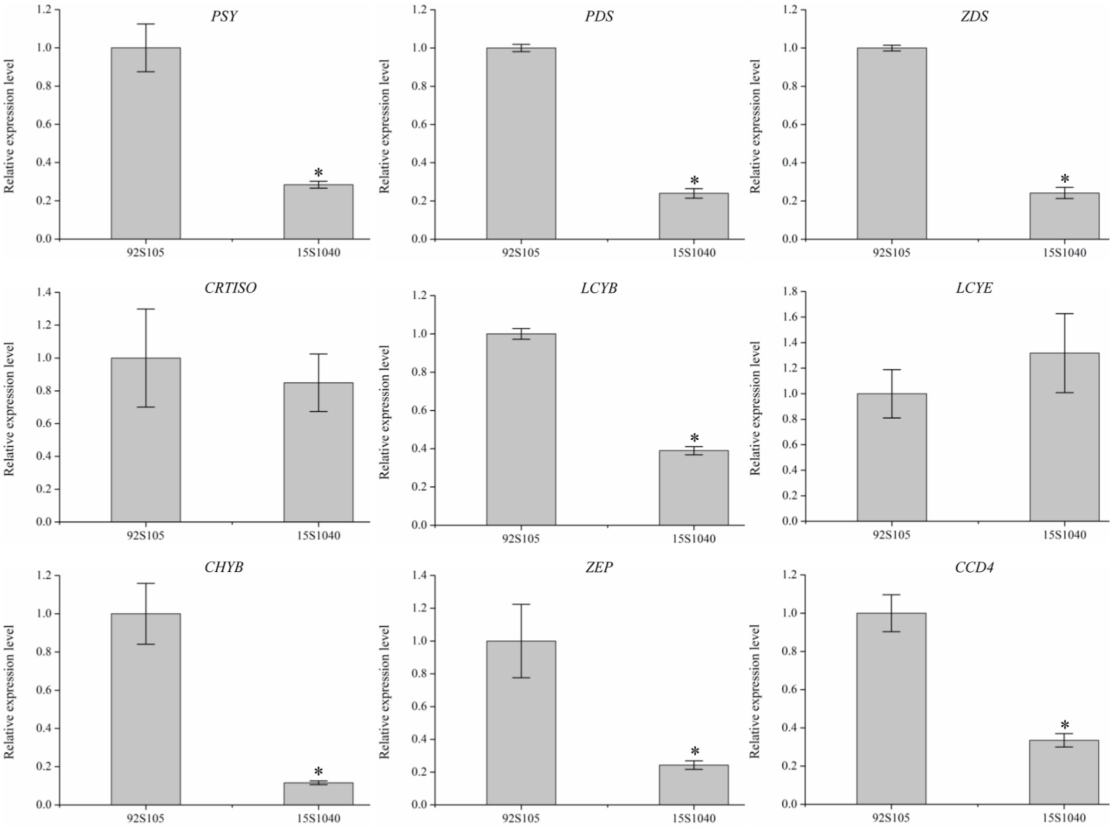

Supplementary Figure S5. Expression analysis of carotenoid metabolic genes in anthesis petals of 92S105 and 15S1040. Error bars indicate the SD, and asterisks represent significant difference (t-test, P < 0.05) between the two parental lines. The expression of the genes in 92S105 was used as the standard of 'relative' expression.
